# A Polytherapy Strategy Using Vincristine and ALK Inhibitors to Sensitise EML4-ALK-Positive NSCLC

**DOI:** 10.3390/cancers14030779

**Published:** 2022-02-02

**Authors:** Josephina Sampson, Hyun-min Ju, Ji-young Song, Andrew M. Fry, Richard Bayliss, Jene Choi

**Affiliations:** 1 Astbury Centre for Structural Molecular Biology, Faculty of Biological Sciences, University of Leeds, Leeds LS2 9JT, UK; i.sampson@leeds.ac.uk; 2 Department of Pathology, Asan Medical Center, University of Ulsan College of Medicine, Seoul 138-736, Korea; yskx24@naver.com (H.-m.J.); 77517751@hanmail.net (J.-y.S.); 3 Department of Molecular and Cell Biology, University of Leicester, Leicester LE1 9HN, UK; andrew.fry@leicester.ac.uk

**Keywords:** EML4-ALK, ALK tyrosine kinase inhibitors, vincristine, microtubules, acetylation, NSCLC, cancer

## Abstract

**Simple Summary:**

Lung cancer is the third most common cancer worldwide with poor survival after initial diagnosis. A large proportion, approximately 85%, of patients with lung cancer are classified as non-small cell lung cancer (NSCLC). The most common genetic alterations in NSCLCs are KRAS, EGFR and ALK mutations. Patients carrying the EML4-ALK mutation are treated with potent ALK drugs. Up to date, there are 15 different EML4-ALK mutations found in NSCLC patients. Of note, patients carrying the EML4-ALK V3 mutation respond poorly to ALK chemotherapeutic regime and acquire resistance. The aim of the present study was to assess the potential of a combination chemotherapy using vincristine, a traditional chemotherapeutic drug, and potent ALK drugs. We demonstrated that vincristine sensitises cells carrying EML4-ALK V1 mutation but not V3. Cells carrying the EML4-ALK V3 mutation confer low response to drug combination due to high levels of tubulin acetylation and active proliferation pathways compared to V1 cells.

**Abstract:**

The oncogenic fusion of EML4-ALK is present in about 4–6% of non-small cell lung cancer (NSCLC). A targeted approach with ALK tyrosine kinase inhibitors (TKIs) has been proven highly effective in ALK-positive NSCLC patients. However, despite the initial responses, the outcome of the treatment is variable. Previous studies have shown that the differential response depends in part on the type of EML4-ALK variant. Here, we examined the combination of ALK inhibitors and microtubule poison, vincristine, in cells expressing EML4-ALK V1 and V3, the two most common variants in NSCLC. We showed that combination therapy of ALK-TKIs with vincristine had anti-proliferative effects and blocked RAS/MAPK, PI3K/AKT and JAK/STAT3 signalling pathways in EML4-ALK V1 but not V3 cells. Our results demonstrate that high levels of tubulin acetylation are associated with poor response to vincristine in EML4-ALK V3 cells. Additionally, we demonstrated differences in microtubule stability between the two EML4-ALK fusions. EML4-ALK V3 cells exhibited dynamic microtubules that confer poor response to vincristine compared to V1 cells. Hence, we suggested that the portion of EML4 in the fusion has an important role for the outcome of the combination treatment.

## 1. Introduction

Lung cancer is one of the most difficult cancers to treat due to later stage diagnosis and complexity of the disease with <20% 5-year overall survival after initial diagnosis [1]. Lung cancers are classified into two histological groups: small cell lung cancers (~15%) and non-small cell lung cancers (NSCLCs, ~85%), of which lung adenocarcinoma and lung squamous cell carcinoma are the major subgroups. The most common genetic alterations in NSCLCs are KRAS and EGFR mutations, and anaplastic lymphoma kinase (ALK) and ROS1 rearrangement [2].

ALK encodes a 1620 amino acid tyrosine kinase comprised of an extracellular domain with ligand binding motifs, a transmembrane helix and a cytoplasmic tyrosine kinase domain [3]. ALK functions are still unclear, although evidence suggests function in the development of the nervous system [4]. Since its discovery as a fusion partner with nucleophosmin (NPM) in anaplastic large-cell lymphoma [5], several other ALK fusions have been identified in a variety of cancers. The most predominant fusion partner of ALK-rearranged NSCLC is the echinoderm microtubule-associated protein (EMAP)-like 4 (EML4) gene. EML4-ALK is present in 2–9% of NSCLC patients and multiple variants of EML4-ALK fusion have been identified. EML4-ALK fusion proteins all contain the ALK kinase domain (exons 20–29 in the ALK gene) but have variable truncations of EML4 (occurring at exons 2, 6, 13, 14, 15, 18 and 20), and the most common variants in patients are variant 1 (V1, 33%), variant 2 (V2, 10%) and variant 3 a/b (V3 a/b, 29%) [6].

EML4 is a member of a family of proteins of which there are six distinct members in humans [7]. EML proteins are localised to and control the dynamics of microtubules, which are essential for the formation of the interphase microtubule network and mitotic spindle. EML4 has an important role in the stabilisation of neuronal microtubules [8]. All EML4-ALK fusion variants have the N-terminal trimerization domain (TD) and some also contain the basic region of EML4, which together are essential for microtubule binding [9]. Recently, it has been reported that the ALK inhibitors, ceritinib and lorlatinib, sequester inactive EML4-ALK V3 protein to microtubules, whereas V1 remains in the cytoplasm [10]. The C-terminal TAPE domain of EML4 consists of a set of 14 individual sub-domains that form a tandem pair of 7-bladed beta-propellers [11]. The presence of the 12N blade subdomain in EML4-ALK V1 protein, which is missing from V3, prevents the catalytically inactive protein from binding to microtubules [6,10].

ALK tyrosine kinase inhibitors (ALK-TKIs), including crizotinib (first-generation), and ceritinib and alectinib (second-generation), have higher objective response rates (ORRs) and progression-free survival (PFS) compared to cytotoxic therapy in ALK-positive patients [12]. However, patient responses to ALK-TKIs are different: the median PFS was shorter among patients with V3 than V1 treated with crizotinib or second-generation ALK inhibitors [13]. Of note, EML4-ALK V3 is significantly associated with higher incidence of ALK resistance mutations, particularly G1202R and metastasis [14]. The third-generation ALK TKI, lorlatinib, showed greater activity towards EML4-ALK V3 NSCLCs containing ALK inhibitor resistance mutations [14].

In the present study, we demonstrate the vulnerability of EML4-ALK V1, but not V3, cells to the microtubule poison, vincristine, when used in combination with the ALK-TKIs, crizotinib and ceritinib. Moreover, our data show that EML4-ALK V3 cells exhibited higher levels of acetylated tubulin and formed more stable microtubules than in V1 cells. The JAK/STAT3 and RAS/MAPK pathways were constitutively active after combination drug treatments in EML4-ALK V3 but not V1 cells. We suggest that these characteristics contribute to a complex resistance mechanism against vincristine treatment in EML4-ALK V3 cells. Hence, the structural differences between the EML4-ALK variants are likely to determine the outcome of this combination treatment.

## 2. Materials and Methods

### 2.1. Cell Culture, Transfection and Drug Treatments

The human NSCLC cancer cell lines, H3122, H2228, H1975, H596, Calu6 and HCC78, were maintained in RPMI 1640 (Invitrogen-Gibco, Carlsbad, CA, USA) containing 10% foetal bovine serum, penicillin (50 μg/mL) and streptomycin (100 μg/mL) (Invitrogen-Gibco) at 37 °C in a humidified 5% CO_2_ incubator. HeLa were transiently transfected using Fugene HD (Promega, UK) according to manufacturer’s instructions. Vincristine, paclitaxel and ceritinib were purchased from Selleckchem (Houston, TX, USA), Crizotinib was provided by Pfizer (Manhattan, NY, USA) (Table S1). Stock solutions were prepared in DMSO and stored at −20 °C. Unless otherwise indicated, the following compounds were added to cells for 4 h: Ceritinib (500 nM); vincristine (20 nM); the compounds were diluted in a fresh media before each experiment and the final concentration of DMSO was <0.1%.

### 2.2. Cell Viability Assay

Cells were seeded in a complete growth medium in 96-well plates at 3 × 10^3^ cells per well. After 24 h, the cells were incubated with crizotinib, ceritinib, vincristine or DMSO in the presence of 10% of FBS. After 72 h of treatment, cell viability was determined using the CellTiter-Glo^®^ Luminescent Cell Viability Assay (Promega, Madison, WI, USA). The half-maximal inhibitory concentration (IC_50_) values were calculated from dose-response curves in the Prism 9.0 software (GraphPad, San Diego, CA, USA).

### 2.3. Cell Extracts and Western Blot Analysis

Whole-cell lysates were prepared in RIPA lysis buffer (Cell Signaling Technology, Danvers, MA, USA) containing a protease inhibitor cocktail (Tech & Innovation^TM^, Bucheon, Korea) and a phosphatase inhibitor cocktail (45065; Santa Cruz, CA, USA). Proteins were separated on an 8% or 10% SDS-PAGE gel, transferred to polyvinylidene fluoride (PVDF) membranes using an iBlot^TM^ dry blotting system (Invitrogen). Immunoblot analysis were performed with the following primary antibodies: pALK (Y1604) (1:1000, 3342; Cell Signaling Technology), ALK (1:1000, 3791; Cell Signaling Technology), pSTAT3 (Y705) (1:1000, 9145; Cell Signaling Technology), STAT3 (1:1000, 9139; Cell Signaling Technology), pAKT (S473) (1:1000, 9271; Cell Signaling Technology), AKT (1:1000, 9272; Cell Signaling Technology), pERK (T202/Y204) (1:1000, 9101; Cell Signaling Technology), ERK (1:1000, 9102; Cell Signaling Technology), Acetyl-α-tubulin (Lys40) (1:1000, T7451; Sigma-Aldrich), GAPDH (1:2000, ab37168; abcam) and β-actin (1:10,000, 5441; Sigma-Aldrich, St. Louis, MO, USA) (Table S2). Secondary antibodies were rabbit or mouse horseradish peroxidase (HRP)-labelled secondary antibodies (1:10,000; Amersham, UK). The blots were visualised using the SuperSignal West Pico Chemiluminescent Substrate (Thermo Fisher Scientific-Pierce, Rockford, IL, USA). Source data from Western blot is shown in Figure S5.

### 2.4. Enzyme-Linked Immunosorbent Assay (ELISA)

Cells were treated with crizotinib (400 nM), ceritinib (400 nM), vincristine (20 nM) alone or combination for 3 h and then lysed with RIPA Buffer (Cell Signaling Technology) supplemented with phosphatase inhibitors (Santa Cruz). ELISA was performed using the following ELISA Kits according to the manufacturers’ instructions: p-ALK (Y1064) (7324CA, Cell Signaling Technology), p-STAT (Y705) (85-86102, Invitrogen), p-AKT (S473) (KHO0111, Invitrogen) and p-ERK (T202/Y204) (ADI-900-098A, Enzo, Farmingodale, NY, USA).

### 2.5. Apoptosis Assay

Annexin V apoptosis assay (FITC Annexin V, Biolegend) was performed according to manufacturer’s instructions. Cells seeded in 12-well plates were treated either with either DMSO, vincristine (20 nM), ceritinib (500 nM) or in combination for 48 h. Cells were harvested and collected by centrifugation. About 5 × 10^5^ cells were incubated in 100 µL Annexin binding buffer, 5 µL Annexin V-FITC and 10 µL propidium iodide (PI) for 15 min on ice. Samples were kept in the dark and diluted in a further 400 µL Annexin binding buffer before analysis by flow cytometry. Samples were processed on a CytoFLEX S flow cytometer (Beckman Coulter) and analysed using Kaluza (Beckman Coulter).

### 2.6. Indirect Immunofluorescence Microscopy

Cells grown on acid-etched glass coverslips were washed with PBS and fixed with 3.7% formaldehyde in PBS buffer for 10 min. Cells were kept in blocking buffer (3% BSA in PBS) for 1 h and incubated for 2 h or overnight with primary antibodies diluted in 3% BSA/PBS buffer followed by 1 h incubation with secondary antibodies. Primary antibodies were against α-tubulin mouse (1:1000, T5168; Sigma-Aldrich), α-tubulin rabbit (1:800; 15246; Abcam, Cambridge, UK), Acetyl-α-tubulin (1:2000, T7451; Sigma-Aldrich), GFP (1:1000, sc-9996; Santa Cruz-Biotechnology) and ALK (D5F3) (1:100, 3633; Cell signalling Technology) (Table S2). Secondary antibodies were Alexa Fluor-488 and -594 anti-rabbit and anti-mouse goat IgGs (1:200, A32723; A32740; Invitrogen). Imaging was performed on a Zeiss LSM880 + Airyscan Inverted confocal microscope using a 40× oil objective (numerical aperture, 1.4). Z-stacks comprising of 10–20 × 0.3 µm sections were acquired. Images were analysed using ImageJ (v.2.0.0).

### 2.7. Microtubule Nucleation Assay

Cells were treated with ceritinib (500 nM), vincristine (20 nM) alone or in combination for 4 h. After treatment, warm media was replaced with cold media, and cells were placed on ice for 30 min to depolymerise microtubules. Cells were then released by changing to warm media for 5 min to allow re-growth of microtubules followed by methanol fixation. The diameter of microtubule nucleation was measured using maximum intensity projections of images for each experimental repeat. The diameter of microtubule nucleation site was measured from 30 cells in each condition. A straight line was drawn across the longest microtubule through the centre of each nucleation site and the diameter was calculated using ImageJ (v.2.0.0) software. Data represent the diameter (μm) of each microtubule nucleation site in each cell from three biological replicates ± SD.

### 2.8. Intensity Measurements of Acetylated Tubulin

Intensity of acetylated tubulin from interphase cells was measured using maximum intensity projections obtained using constant exposure times and gain settings for each experimental repeat. Total of 20–30 images were analysed for acetylated tubulin intensity measurements. The intensities were measured using Colo2 in ImageJ (v.2.0.0) software. A 100 pixels × 100 pixels ROI was positioned over the interphase cell and intensity was measured in each treatment. Data represent the percentage of intensity of acetylated tubulin from at least two independent experiments ± SD.

### 2.9. Statistical Analysis

All quantitative data represent means and standard deviation (SD) of at least three biological replicates. Statistical analyses were performed using one-way or two-way ANOVA analysis and student *t*-test from Prism 9.0 software. **** *p* < 0.0001, *** *p* < 0.001, ** *p* < 0.01, * *p* < 0.05. The synergistic effect of ALK TKIs and vincristine was calculated using SynergyFinder 2.0 software.

## 3. Results

### 3.1. NSCLC Cells Expressing EML4-ALK V1 Are Hypersensitive to Vincristine

**Figure 1:**
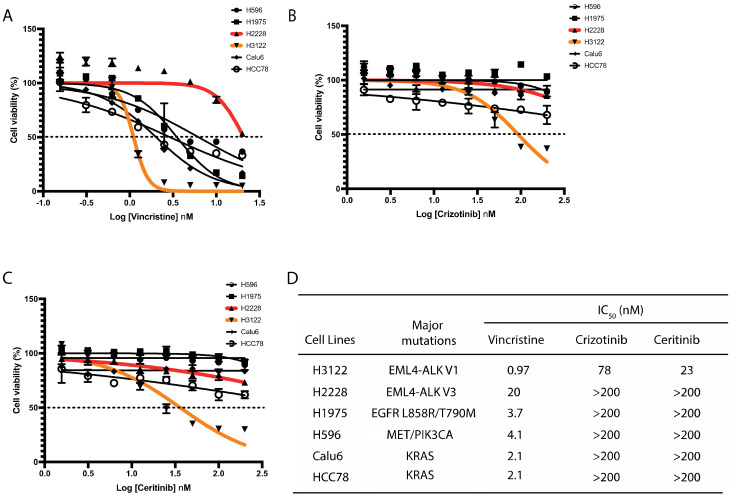
H3122 but not H2228 cells are sensitive to vincristine and ALK-TKIs. (**A**–**C**). Lung cancer cells were treated with increasing doses of vincristine (**A**), crizotinib (**B**) and ceritinib (**C**) for 72 h. Cell viability was determined using CellTiter-Glo assays. The IC_50_ values were calculated using the Prism 9.0 software. Data represent the mean of three biological replicates in each column; the bars denote ±SD. (**D**). Table summarises the IC_50_ of vincristine and ALK inhibitors in lung cancer cell lines harbouring major oncogenic mutations.

Given the importance of EML4 in microtubule stability and the observation that EML4-ALK V3 fusion protein binds to microtubules [9,15], we hypothesised that a microtubule poison such as vincristine will be beneficial for sensitizing those NSCLC cells harbouring EML4-ALK V3 mutation. Vincristine is a microtubule-destabilising inhibitor which blocks tubulin polymerisation. We initially analysed the responses to vincristine of six NSCLC cell lines harbouring distinct genomic alterations including *EML4-ALK*, *EGFR*, *MET*, *PIK3CA* and *KRAS*. Vincristine administered at concentrations of 0.156 to 20 μmol/L (or Log (–1) to Log (1.5) nM) led to significant growth suppression of NSCLC cells with a wide range of IC_50_ values. H3122 cells (EML4-ALK V1) displayed the highest sensitivity to vincristine (Figure 1A,D). In contrast, H2228 cells (EML4-ALK V3) showed the most resistance of all lung cancer cell lines examined to vincristine with around 20-fold higher IC_50_ compared with H3122 cells (Figure 1A,D). Interestingly, paclitaxel, a microtubule-stabilising agent, is potently cytotoxic on H2228, H3122 and other NSCLC cell lines (Figure S1A,B). We next examined ALK-TKI responses on these six lung cancer cell lines. As expected, H3122 cells displayed the greatest degree of sensitivity to crizotinib or ceritinib, but the other lung cancer cells lines, including H2228, did not show cytotoxic response (Figure 1B–D). These results indicate that H3122 and H2228 cells have different sensitivities to vincristine and ALK-TKIs.

### 3.2. Growth-Inhibitory Effect of ALK-TKIs and Vincristine Combination in H3122 but Not H2228 Cells

**Figure 2:**
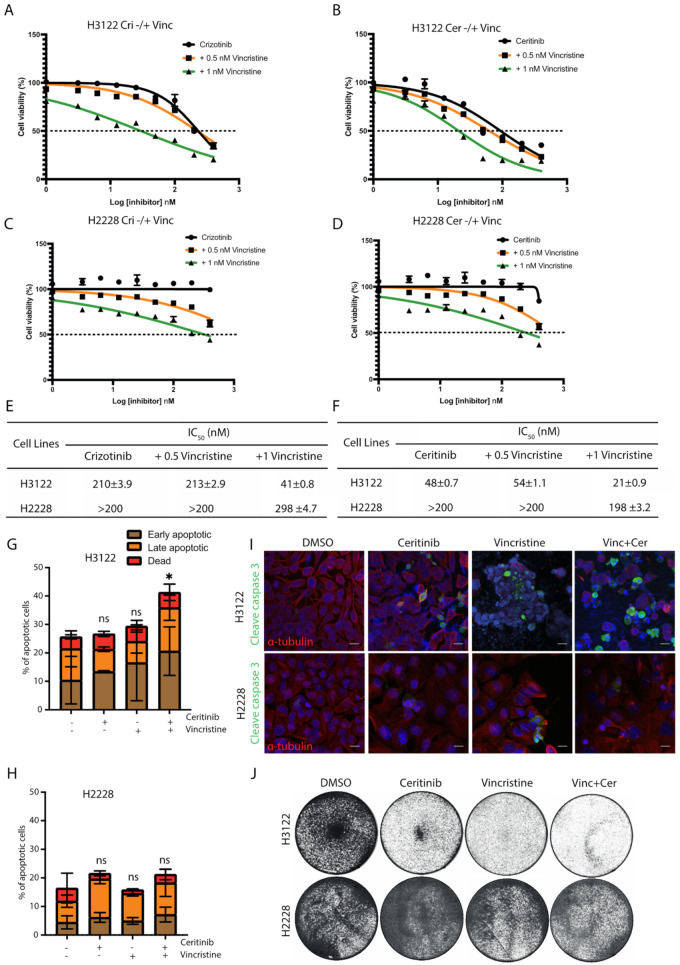
The combination of vincristine and ALK inhibitors enhances the anti-proliferative effect in EML4-ALK V1 cells, but not in V3. ALK-positive lung cancer cells, H3122 (V1) and H2228 (V3), were treated with increasing doses of (**A**,**C**) crizotinib −/+ vincristine (0.5 or 1 nM), or (**B**,**D**) ceritinib −/+ vincristine (0.5 or 1 nM) for 72 h. Cell viability was determined using CellTiter-Glo assays. The IC_50_ values were calculated using the Prism 9.0 software. Data represent the mean of three biological replicates in each column; the bars denote ±SD. (**E**,**F**) Table summarises the IC_50_ of the combination of vincristine and ALK inhibitors in H3122 and H2228 cells. (**G**,**H**) H3122 and H2228 cells were treated with either vincristine, ceritinib or in combination for 48 h before analysis by annexin V-based flow cytometry. Histograms represent the percentage of cells in apoptosis. Cells were classified as early apoptotic, late apoptotic and dead. Data represent the mean of three biological replicates; the bars denote ±SD. * *p <* 0.5 in comparison to DMSO by two-way ANOVA. (**I**) H3122 and H2228 cells were treated with either single agents of vincristine or ceritinib or in combination for 24 h before being fixated and stained with cleaved caspase-3 (green), anti-α-tubulin (red), and DAPI (blue). Scale bars, 20 μm. (**J**). Crystal violet staining of H3122 and H2228 cells. Cells were treated with the indicated drugs for 72 h before fixed and stained. ns= not significant.

Since EML4-ALK V3, but not V1, is sequestered to microtubules in the presence of ALK-TKIs [10], we tested whether the addition of vincristine could enhance the cytotoxicity of ALK-TKI (crizotinib or ceritinib) in ALK-positive lung cancer cell lines. The combination of crizotinib plus vincristine (1 nM) was more effective in reducing cell viability than crizotinib alone with ~5-fold lower IC_50_ in H3122 cells (Figure 2A). The ceritinib and vincristine (1 nM) combination was also more effective in H3122 cells with ~2-fold lower IC_50_ values, compared with ceritinib alone (Figure 2B). 

Although co-treatment with either crizotinib or ceritinib plus vincristine resulted in decreased cell viability in H2228 cells, these cells were substantially less sensitive to the combination than H3122 cells (Figure 2C–F). To investigate the sensitisation between vincristine and ALK-TKI (crizotinib and ceritinib) combinations, we analysed the data using SynergyFinder. Scores of >0 show a synergistic effect, whereas scores of <0 indicate an additive effect. Data analysis revealed no evidence of synergy in H2228, and only some evidence of weak synergy in H3122 (Figure S2E–H).

We next assessed whether this pronounced effect observed with vincristine and ceritinib treatment was a result of apoptotic cell death. Ceritinib or vincristine alone or in combination led to a ~2-fold increase in apoptosis in H3122 cells, assessed via Annexin V+PI staining (Figure 2G). In contrast, low levels of cell death were observed in single and combination treatments with ceritinib and vincristine in H2228 cells (Figure 2H). In addition, combination treatments led to a significant expression of cleaved caspase-3, an apoptotic protein, in H3122 cells, but not in H2228 (Figure 2I and Figure S2A,B). Treatment of H3122 cells with ceritinib or vincristine led to a near-complete inhibition of cell viability assessed by crystal violet staining, but no change in H2228 cells (Figure 2J). These data suggest that the combination of vincristine and ALK-TKIs is more effective in eliciting apoptotic cell death in H3122 cells than in H2228. We next investigated the mechanism of resistance to this combination in the H2228 cells, starting with the functional consequence of EML4-ALK V3 localisation to microtubules.

### 3.3. H2228 Cells Harbouring EML4-ALK V3 Exhibit Robust Microtubule Nucleation

**Figure 3:**
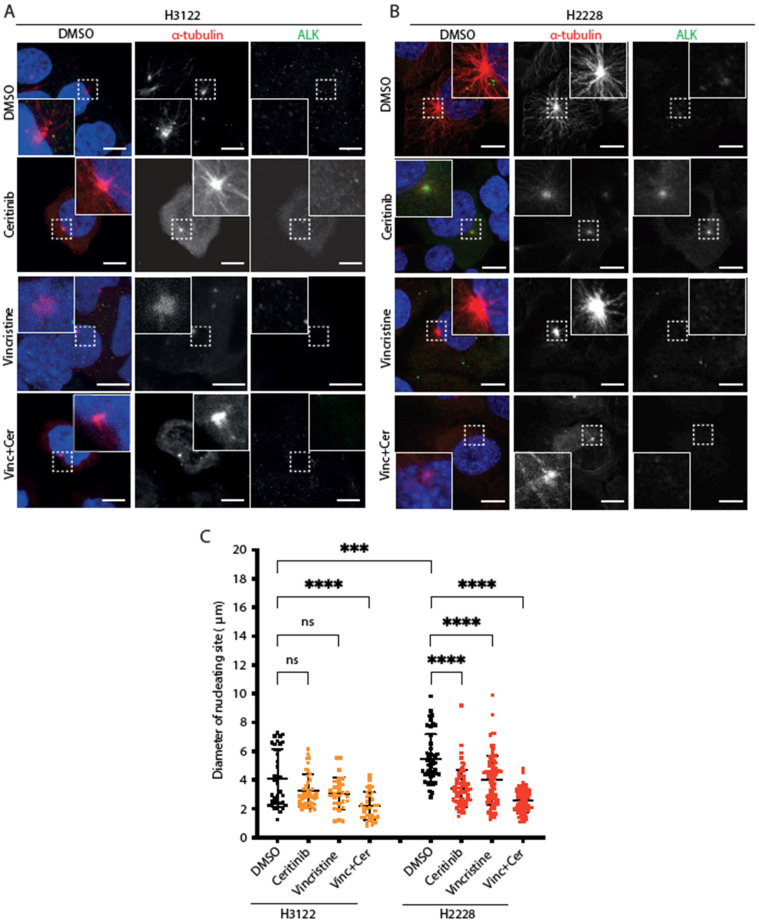
H2228 cells have higher microtubule nucleation capacity than H3122 cells. (**A**,**B**). H3122 and H2228 cells were treated with either DMSO, vincristine, ceritinib or in combination for 4 h, then incubated on ice for 30 min to depolymerise microtubules. The cells were released into warm medium for microtubule reconstruction for 5 min before being fixed and stained with anti-ALK (green), anti-α-tubulin (red), and DAPI (blue). Scale bars, 10 μm; magnified views of a selected area are shown. (**C**). Dot plot shows the diameter of nucleating site per cell from (**A**,**B**). Data represent counts from >20 cells, *n* = 3. *** *p <* 0.001, **** p < 0.0001 in comparison to DMSO by one-way ANOVA. ns= not significant.

Based on the previous observations, we examined microtubule nucleation activity in cells expressing EML4-ALK variants after vincristine, ceritinib or combination treatments. For this purpose, cells were placed on ice for 30 min to depolymerise microtubules followed by analysis of microtubule re-growth upon addition of warm media (Figure 3A,B). After a 5 min release in warm medium, the diameter of the microtubule aster was significantly larger in H2228 compared to H3122 cells in the absence of drug treatment (Figure 3C). Treatment with ceritinib or vincristine alone suppressed microtubule nucleation in H2228 but not H3122 cells (Figure 3C). The combination of ceritinib and vincristine led to a significant reduction of microtubule nucleation in both H3122 and H2228 cells (Figure 3C). Thus, H2228 cells that harbour EML4-ALK V3 have the capacity to more rapidly generate microtubules than H3122 cells that harbour EML4-ALK V1, and this capacity is reduced by ALK inhibition, a microtubule poison that decreases polymerisation, or both. However, the combination significantly decreased microtubule nucleation capacity in both H3122 and H2228 cells, suggesting that other molecular mechanisms must contribute to the different overall sensitivity of the two cell lines to the combination.

### 3.4. High Microtubule Acetylation of H2228 Cells Is Correlated with Low Response in Vincristine Treatment

**Figure 4:**
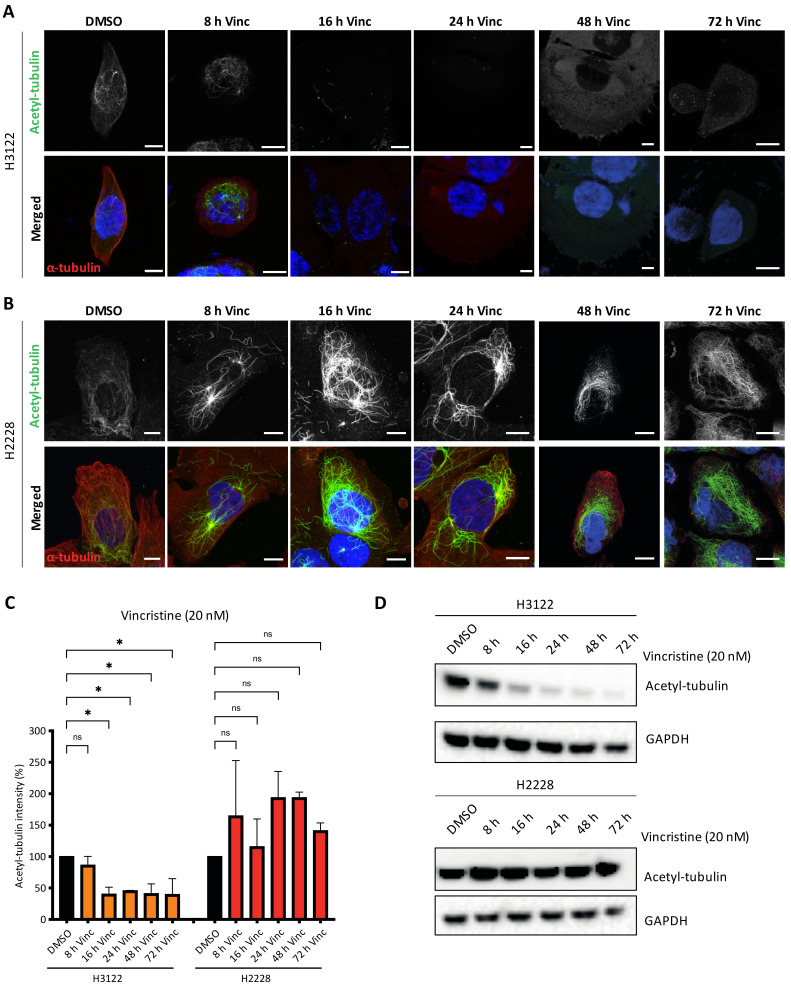
Vincristine treatment reduces tubulin acetylation in H3122, but not in H2228 cells. (**A**,**B**). H3122 and H2228 cells were treated with vincristine (20 nM) at the indicated time points before fixated and stained with anti-acetylated tubulin (green), anti-α-tubulin (red) and DAPI (blue). Scale bars, 10 μm. (**C**). Box plot shows the intensity of acetylated tubulin from (**A**,**B**). Data represent counts from >20 cells, *n* = 2. (**D**). H3122 and H2228 cells were treated with vincristine at the indicated time points. Western blots of acetylated tubulin are shown. GAPDH was used as a loading control. * *p* < 0.05, ns = not significant.

Previous studies reported that EML4-ALK V3 proteins promote microtubule stabilisation and increased microtubule acetylation compared to V1 [16]. Acetylation of α-tubulin on lysine 40 (K40) is the only known post-translation modification within the lumen of microtubules. It is found in stable and long-lived microtubules with this modification facilitating microtubule self-repair and protection from mechanical breakage or depolymerising drugs [17]. We hypothesised that H2228 cells harbouring EML4-ALK V3 may have high levels of acetylated microtubules since EML4 binds and stabilises microtubules. To address this hypothesis, we treated H3122 and H2228 cells with vincristine, then evaluated the levels of K40 acetylation after 8, 16, 24, 48 and 72 h (Figure 4A,B). Acetylated microtubules levels showed a two-fold decrease in vincristine-treated H3122 cells, while vincristine-treated H2228 cells showed the opposite effect with a two-fold increase of acetylated MTs (Figure 4C). Similarly, vincristine treatment altered the acetylated tubulin levels in overexpressed YFP-EML4-ALK V1, but not V3, HeLa cells (Figure S3A–C). This behaviour was confirmed by immunoblotting with acetylated tubulin (K40) antibodies, indicating that vincristine reduced K40 acetylated tubulin levels in H3122 cells, but not in H2228 (Figure 4D and Figure S3D). The persistence in acetylated tubulin in H2228 cells treated with vincristine, when compared to H3122 cells may explain their lower sensitivity to this microtubule poison.

### 3.5. The Combination of ALK-TKI and Vincristine Inhibits Multiple Signalling Pathways

**Figure 5:**
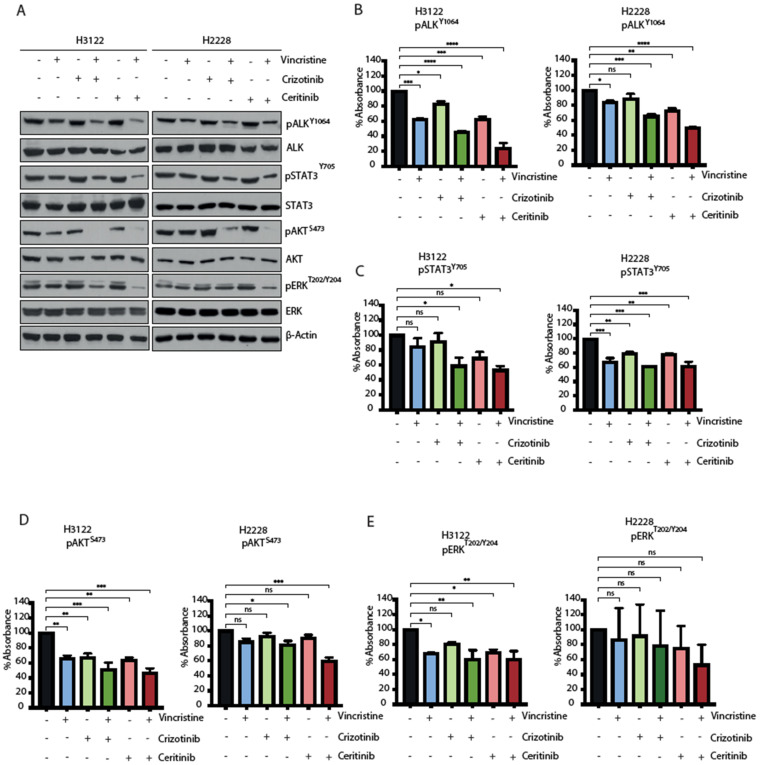
The combination of ALK inhibitor and vincristine impairs downstream signalling of RAS/MAPK, PI3K/AKT and JAK/STAT3 in H3122 but not H2228 cells. (**A**). H3122 and H2228 cells were serum starved overnight and then treated with the indicated inhibitors for 3 h (crizotinib/ceritinib 400 nM and vincristine 20 nM). Western blotting analysis for the indicated proteins was performed. β-actin was used as a loading control. (**B**–**E**). ELISA assay was used to quantify expressions of pALK^Y1604^, pSTAT3^Y705^, pAKT^S473^ and pERK^T202/Y204^ proteins. Data represent counts from three biological replicates, *n* = 3. * *p <* 0.05, ** *p <* 0.01, *** *p <* 0.001, **** p < 0.0001 in comparison to DMSO by one-way ANOVA. ns = not significant.

EML4-ALK proteins drive oncogenic signalling via PI3K-AKT, MAPK and JAK-STAT pathways [18,19]. We next examined how the ALK-TKI and vincristine combination affects signalling pathways in H3122 and H2228 cells. EML-ALK-positive cells were treated with a single agent or in combination for 48 h and the consequences for signalling was evaluated for ALK (pY1604), STAT3 (pY705), AKT (pS473) and ERK (pT202/Y204) through immunoblotting and ELISA-based assays (Figure 5A–E and Figure S4A). The combination of vincristine and crizotinib led to a more significant reduction than single compounds in the signal of all the phosphorylation markers in H3122 cells, but only ALK (pY1604) and AKT (pS473) in H2228. The combination with vincristine and ceritinib was significantly more effective overall and led to a decrease in ALK (pY1604) and AKT (pS473) phosphorylation in both cell lines. Analysis of immunoblot and ELISA assays also showed a significant reduction of ERK (pT202/Y204) signal in H3122 cells, and the immunoblot data also showed reduced signal in H2228 cells. In contrast, the combination of vincristine and ceritinib only showed a significant reduction of STAT3 (pY705) compared to individual treatments in the H3122 cells, not the H2228 cells. Taken together, these data suggest that the combination of ALK-TKI and vincristine is more effective against PI3K/AKT signalling pathway in both H3122 and H2228 cells, but not against MAPK/ERK and JAK/STAT3 in H2228 cells.

## 4. Discussion

There is an urgent need to explore new ways to tackle progression and resistance in ALK-positive NSCLC and especially the ones that harbour EML4-ALK V3 mutation. In this paper, we explored new ways to target ALK-positive NSCLC cell lines by using traditional chemotherapeutic drugs such as the vinca alkaloid, vincristine, in combination with ALK-TKIs. We have shown that vincristine either alone or in combination with ALK-TKIs sensitises EML4-ALK V1-harbouring cells, but not V3 cells. Specifically, we have shown that EML4-ALK V3 cells exhibit dynamic microtubules compared to V1 cells. We then demonstrated that high levels of acetylated tubulin are correlated with a poor response after microtubule poison and ALK-TKI treatments in the EML4-ALK V3-harbouring cell line H2228.

While EML4 has a profound role in microtubule stability and mitotic spindle organisation, EML4-ALK fusions proteins exhibit different localisation patterns and cellular functions [8,9,15,20]. Experimentally, the short EML4-ALK variant 3 binds to microtubules, whereas the long EML4-ALK variant 1 does not bind due to the partial TAPE domain and 12N blade, even though both variants have the key microtubule binding region [9,11]. In our data, the short EML4-ALK V3 fusion had higher microtubule nucleation capacity compared to the long EML4-ALK V1 protein. This is not surprising, given our recent discovery of a specific region within the partial TAPE domain within the EML4-ALK V1 fusion protein that suppresses microtubule binding [10].

Microtubules are intracellular cytoskeletal components with key roles in cell morphology, migration and signalling. Vincristine, a traditional vinca alkaloid chemotherapeutic drug that destabilises microtubules, is often used in treatment of several cancers. However, long-term treatment with microtubule poisons leads to alterations of microtubule PTMs and tubulin isotype expression [17,21]. Several post-translational modifications (PTMs) of α-tubulin can control cellular functions such as signalling, differentiation, and trafficking [22]. One such important PTM is the acetylation of α-tubulin that occurs on lysine 40 (K40) by the α-tubulin acetyltransferase 1 (α-TAT1) [23,24]. Histone deacetylase 6 (HDAC6) and sirtuin 2 (SIRT2) are negative regulators of α-tubulin acetylation. The relationship between acetylated α-tubulin and drug resistance is still not well understood. However, previous studies reported that high levels of α-tubulin acetylation confer chemoresistance in docetaxel-resistant prostate cancer and paclitaxel-resistant lung cancer cells [17,25]. Metastatic breast cancer cell lines contain high levels of α-tubulin acetylation which promote formation of protrusions and confer a more aggressive phenotype [26].

In line with these studies, we observed high levels of α-tubulin acetylation and resistance to cell death after treatment with vincristine in EML4-ALK V3 but not V1 cells. This suggests a potential role of tubulin acetylation to evade apoptosis. A similar mechanism has been observed in paclitaxel-resistant cells [17]. This could possibly explain why vincristine and vincristine/ALK-TKI combinations were not effective in the treatment of EML4-ALK V3 cells. On the other hand, the EML4-ALK V1 cells responded well to the combination of vincristine and ceritinib treatment, potentially due to lower levels of acetylated α-tubulin. EML4-ALK V3 protein regulates cell morphology and migration as well as microtubule stabilisation through NEK9 and NEK7 kinases, resulting in enhanced cell motility that might contribute to the increased metastasis observed in V3 patients [16,27]. Acetylation of α-tubulin could potentially serve as a marker for invasive and metastatic NSCLC, as well as a potential prognostic indicator.

Crizotinib and ceritinib are clinically used ALK-TKIs that potently inhibit EML4-ALK fusion in NSCLC. As single agents, we showed reduced cell viability in H3122 cells, but not in H2228 as has been previously observed in other studies [13,28]. The combination treatments with vincristine induced greater loss of cell viability than the singe-agent treatment in both cell lines. However, H2228 cells were still less sensitive to the combination than H3122 cells. In addition, all four NSCLC cell lines tested were sensitive to paclitaxel, suggesting that the mechanisms of resistance to vincristine in H2228 cells do not confer resistance to this micro-tubule-stabilising drug. Paclitaxel is therefore of interest in further combination studies. The only clear difference in molecular signalling between these cell lines in response to the vincristine/ALK inhibitor combination treatment was phosphorylation of STAT3 and ERK, which was further reduced in H3122 but not H2228. A few studies have provided evidence that STAT3 regulates microtubule dynamics and antagonises the depolymerizing activity of stathmin [29,30]. Elevated STAT3 levels found across several human cancers and the constitutive STAT3 activity promote cell proliferation, invasion and evasion from cell death [31]. Therefore, the presence of increased STAT3 activity may control microtubule stability and protect EML4-ALK V3 harbouring cells from death after combination drug treatments. In line with our observations, a recent study has reported synergy of the ALK-TKIs, crizotinib, ceritinib and alectinib, with the STAT3 inhibitor, YHO-1701, in a H2228 xenograft mouse model [32]. This suggests that an ALK inhibitor-STAT3 inhibitor polytherapy is a promising approach to tackle EML4-ALK V3 positive-NSCLC cells in the future.

## 5. Conclusions

**Figure 6:**
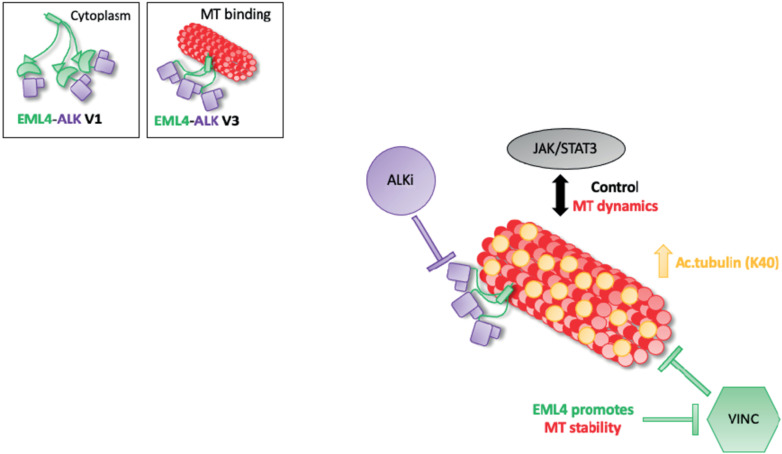
Model summarising the relationship between ALK signalling and MT dynamics in EML4-ALK V3. Schematic model showing the proposed drug resistance mechanism of EML4-ALK V3. EML4-ALK variants, the protein exists as a trimer via the dynamic interactions of the EML4 trimerization domain that drive ALK activation and signalling, but only EML4-ALK V3 associates efficiently with microtubules to enhance stability and confer resistance to vincristine. The inactivation of ALK kinase via ALK-TKIs sequesters EMl4-ALK V3 to microtubules, promoting tubulin acetylation and chemoresistance. Additionally, EML4-ALK V3 maintains an active JAK/STAT3 pathway in the presence of ALK-TKIs. Taken together, these drug-resistance mechanisms allow EML4-ALK V3 to escape cell death.

In conclusion, we have shown that combined ALK inhibitor and microtubule destabilising agent treatment inhibits proliferation in EML4-ALK V1 but not V3-harbouring cells. It is known that microtubule network facilitates cellular signalling and EML4 promotes microtubule stability. In EML4-ALK V3, single or combination treatments increase the levels of acetylated tubulin promoting a stable microtubule network and potentially with the aid of the active JAK/STAT3 pathway prevent cell death (Figure 6). The short EML4-ALK V3 cells exhibited stable microtubules, but not V1, suggesting that the part of EML4 protein in each variant could determine the outcome of the treatment. This study suggests a drug resistance mechanism of EML4-ALK V3, which has a significant impact on both ALK-TKI and vincristine response. However, the EML4-ALK V1 cell line was sensitive to those drug treatments. Overall, these results provide new mechanistic understanding to support rational approaches to polytherapy in EML4-ALK positive patients, in which the specific combinations are chosen based on variant type and other molecular markers such as acetylated tubulin. This strategy is one we believe should be taken to improve the outcomes for EML4-ALK positive-NSCLC patients in the future.

## Data Availability

The data presented in this study are available on request from the corresponding authors.

## Supplementary Materials

The following are available online at https://www.mdpi.com/article/10.3390/cancers14030779/s1. Figure S1: Paclitaxel treatment of NSCLC cell lines. Figure S2: High expression levels of apoptotic proteins upon vincristine and ceritinib treatments in H3122, but not in H2228. Figure S3: EML4-ALK V3 cells, but not V1, express high levels of acetylated tubulin upon vincristine treatment. Figure S4: Vincristine and ALK-TKIs inhibit signalling pathways in EML4-ALK-positive cells. Figure S5: Source data from Western blot Figure 4, Figure 5 and S2. Table S1: Compounds used and concentrations, Table S2: Antibodies used for immunofluorescence (IF) and western blotting (WB) and dilutions.
