# Supplementary material for: A Polytherapy Strategy Using Vincristine and ALK Inhibitors to Sensitise EML4-ALK-Positive NSCLC
Source: Cancers (Basel). 2022 Feb 2;14(3):779. doi: 10.3390/cancers14030779 (PMC8833940; doi:10.3390/cancers14030779)
Supplement: Supplementary file 1 [file cancers-14-00779-s001.zip › cancers-1526055-supplementary.pdf]

TABLES

Table S1. Compounds used and concentrations

Compound	Supplier	Final concentration
Crizotinib	Pfizer	400 nM
Ceritinib	Selleck Chemicals	500 nM
Vincristine	Selleck Chemicals	20 nM
Paclitaxel	Selleck Chemicals	A range of doses

Table S2. Antibodies used for immunofluorescence (IF) and western blotting (WB) and dilutions

Antibody	Supplier	Identifier	IF dilution	WB dilution
Anti-α-tubulin mouse monoclonal	SIGMA-ALDRICH	T5168	1:1000	1:2000
Anti-α-tubulin rabbit polyclonal	Abcam	ab15246	1:800	1:2000
Anti-GFP rabbit polyclonal	Abcam	ab6556	1:1000	1:1000
Anti-GFP mouse monoclonal	Santa Cruz Biotechnology	sc-9996	1:1000	1:1000
Anti-ALK (D5F3) rabbit monoclonal	CST	3633	1:100	1:1000
Anti-ALK (31F12) mouse monoclonal	CST	3791	1:100	1:1000
Anti- phospho ALK (Y1604) rabbit monoclonal	CST	3341		1:1000
Acetyl-α-tubulin mouse	SIGMA-ALDRICH	T7451	1:1000	1:2000
Anti-STAT3 mouse monoclonal	CST	9139		1:1000
Anti-phospho STAT3 (Y705) rabbit monoclonal	CST	9145		1:1000
Anti-AKT rabbit monoclonal	CST	9272		1:1000
Anti-phospho AKT (S473) rabbit monoclonal	CST	9271		1:1000
Anti-ERK rabbit monoclonal	CST	9102		1:1000

Anti-phospho ERK (T202/Y204) rabbit monoclonal	CST	9101		1:1000
Anti- β-Actin mouse monoclonal	Sigma-Aldrich	5441		1:10,000
Anti-GAPDH rabbit monoclonal	Abcam	ab37168		1:2000

A

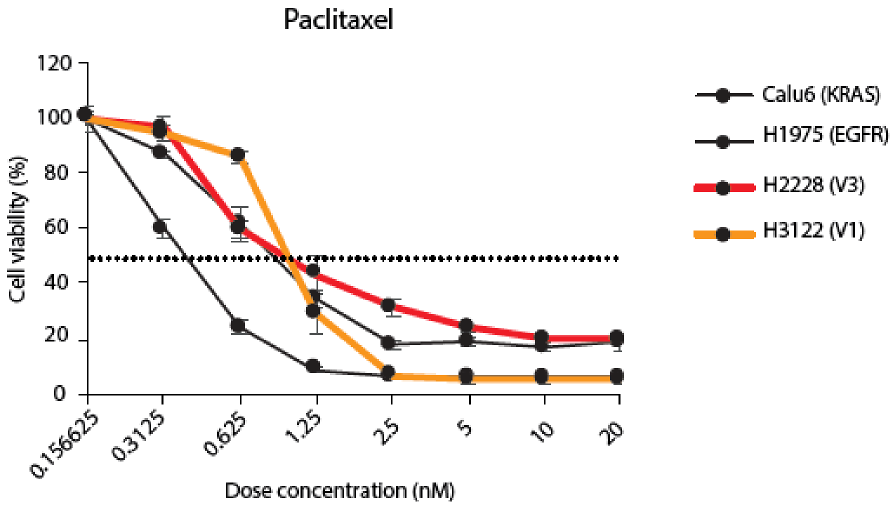

B

Cell Lines	Major mutations	IC_{50} (nM)
		Paclitaxel
Calu6	KRAS	0.97
H1975	EGFR L858R/T790M	0.41
H3122	EML4-ALK V1	1.15
H2228	EML4-ALK V3	1.18

Figure S1. Paclitaxel treatment of NSCLC cell lines

A. NSCLC cell lines harbouring different genetic alterations were treated with increasing doses of paclitaxel for 72 hours. Cell viability was determined using CellTiter-Glo assays. Data represent the mean of three biological replicates in each column; the bars denote ±SD. **B.** Table summarizes the IC_{50} of paclitaxel treatment in NSCLC cell lines.

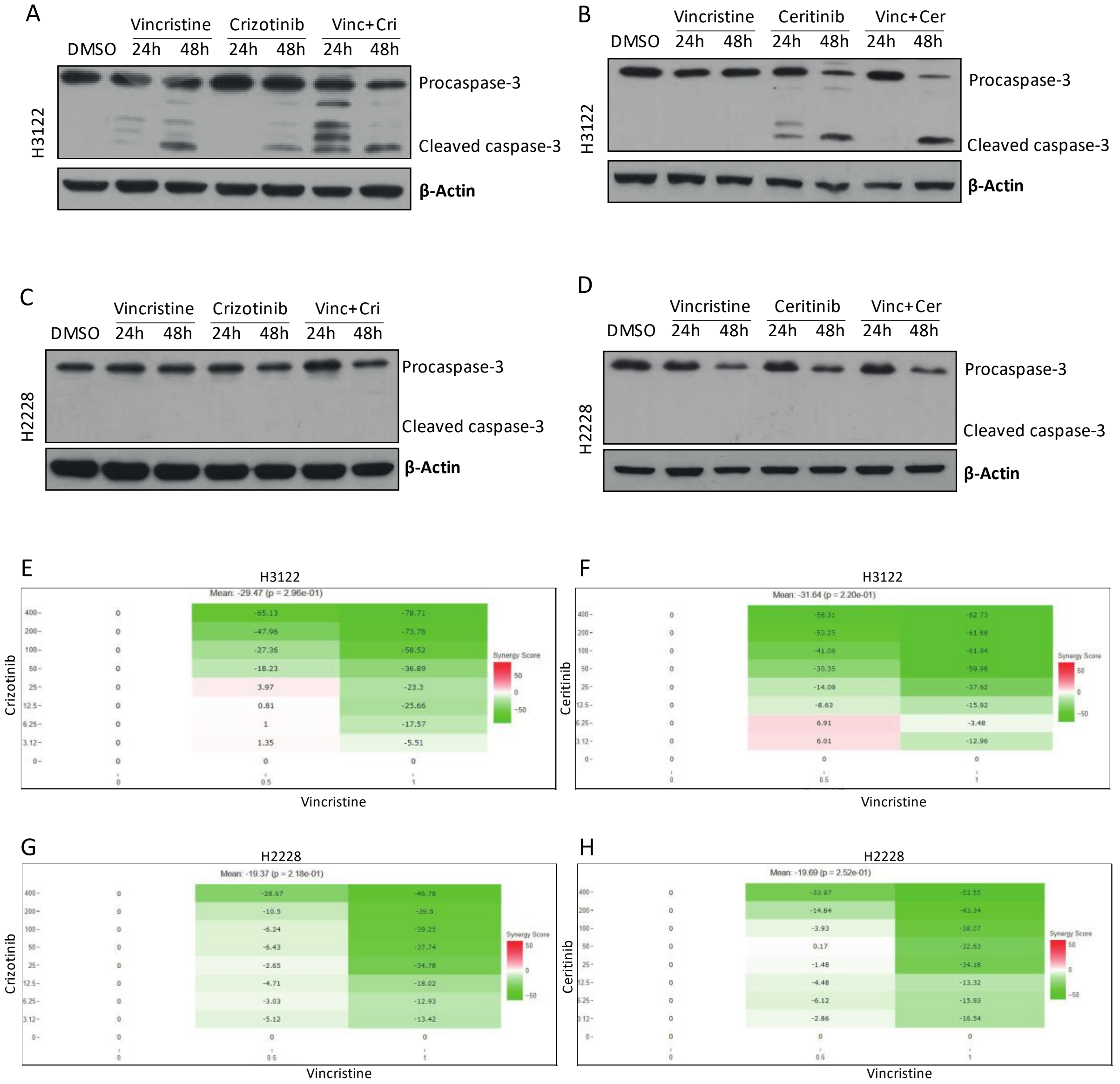

Figure S2. High expression levels of apoptotic proteins upon vincristine and ceritinib treatments in H3122, but not in H2228

A, B. H3122, **C, D.** H2228 cell lines were treated with the indicated drugs (400 nM crizotinib; 400 nM ceritinib; 20 nM vincristine) for either 24 or 48 hours. Western blotting analysis for the indicated proteins was performed. β-actin was used a loading control. **E-H**. Heatmaps summarizing the sensitization effects of vincristine and ALK-TKIs, crizotinib and ceritinib. Data were analysed by SynergyFinder software 2.0. The score of < 0 (green) confers additive effect, whereas the score of > 0 (red) confers sensitivity.

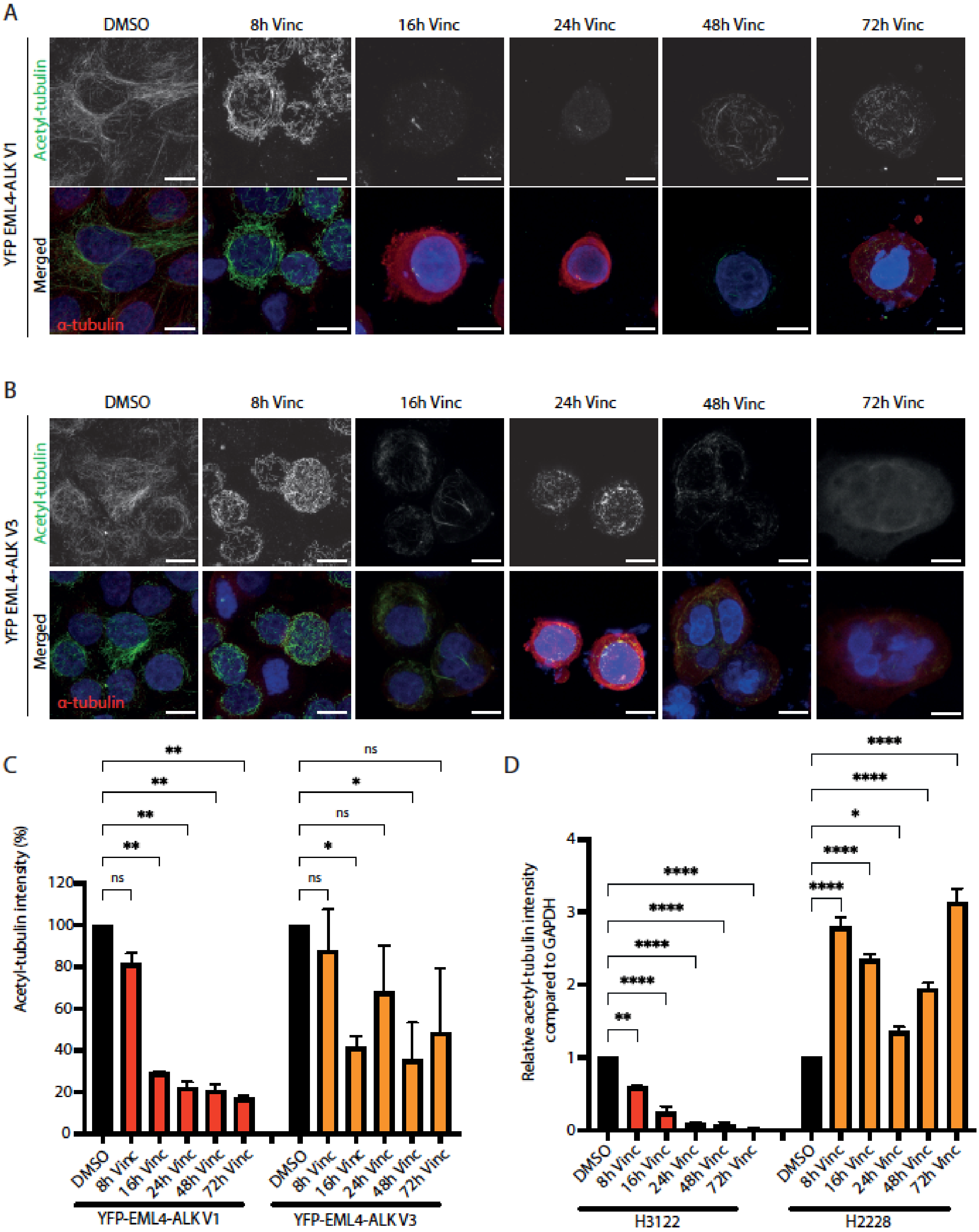

Figure S3. EML4-ALK V3 cells, but not V1, express high levels of acetylated tubulin upon vincristine treatment

A, B. HeLa cells were transfected with either YFP-EML4-ALK V1 or V3 constructs for 48 hours and treated with vincristine at the indicated time points. Cells were fixed and stained with anti-acetylated tubulin (green), anti-α-tubulin (red), and DAPI (blue). Scale bars, 10 μm. **C.** Box plot shows the intensity of acetylated tubulin from A and B. Data represent counts from >15 cells, the bars denote ±SD, *n*= 2. **D.** Acetylated tubulin band intensity was quantified relative to GAPDH from Figure 4D. Box plot graph is representative of two biological replicates, the bars denote ±SD.

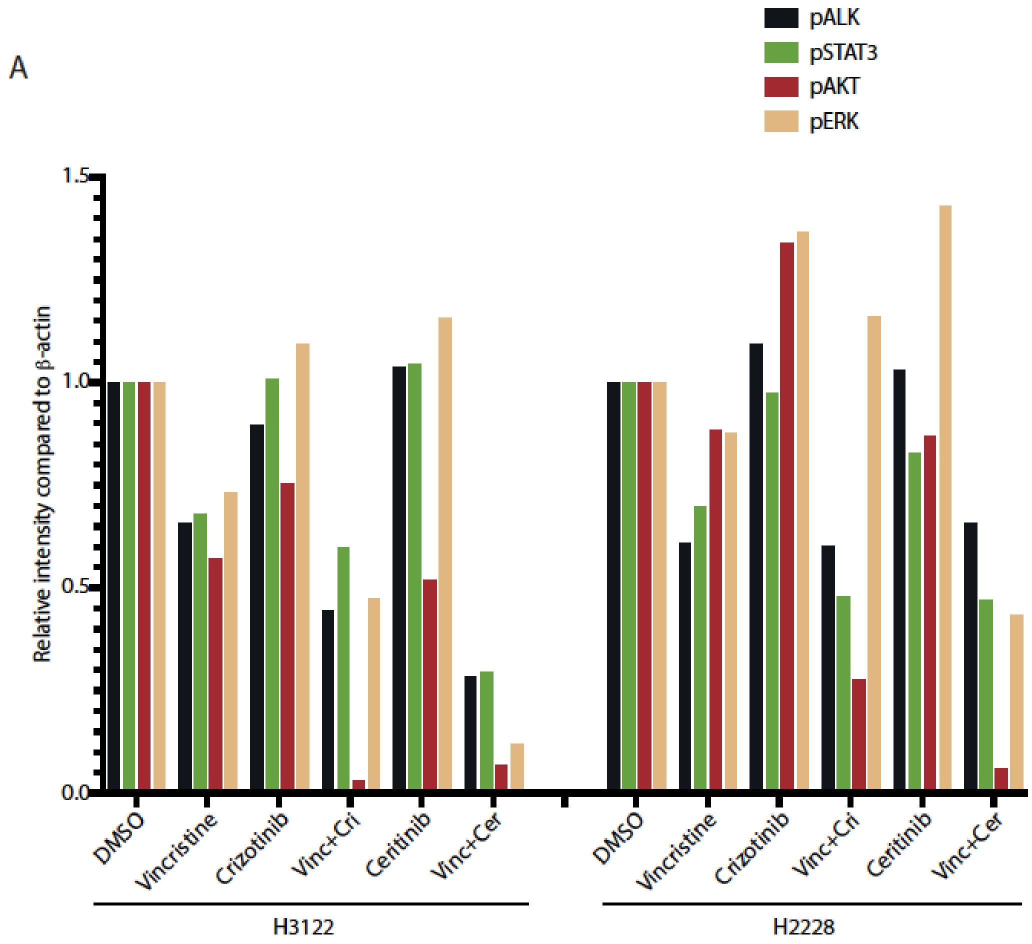

Figure S4. Vincristine and ALK-TKIs inhibit signalling pathways in EML4-ALK-positive cells A. H3122 and H2228 cell lines were treated with the indicated drugs (400 nM crizotinib; 400 nM ceritinib; 20 nM vincristine) for 4 hours. Signalling proteins were quantified relative to β-actin. Box plot graph is representative of western blotting analysis from Figure 5A.

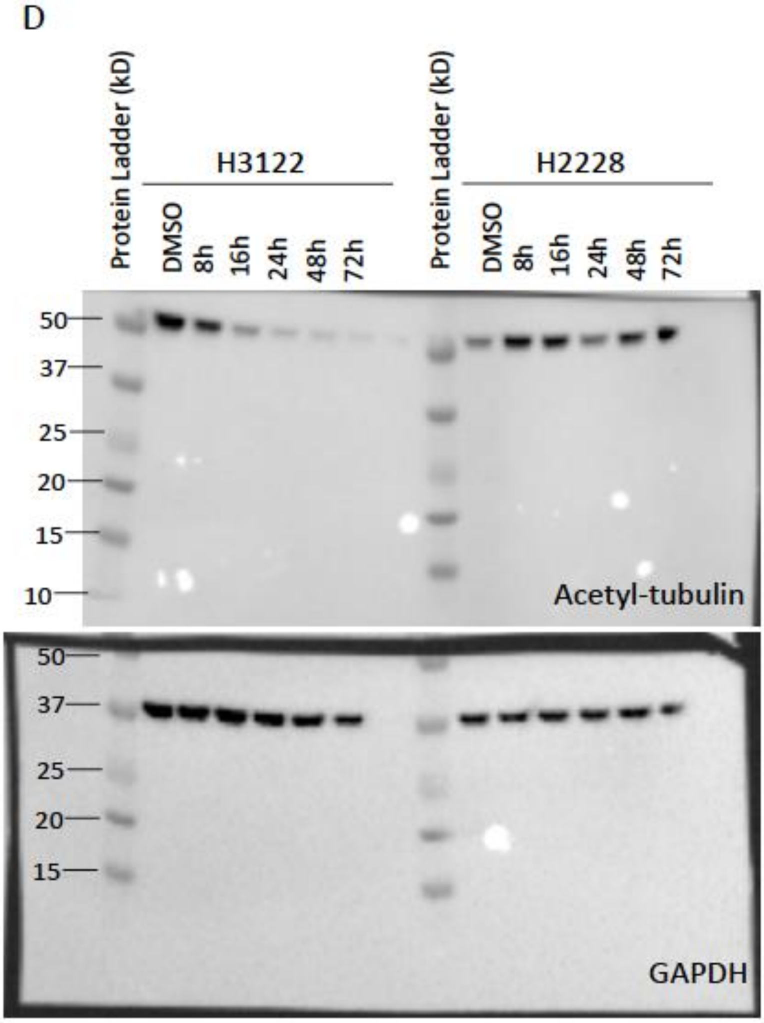

Figure S5. Source data of western blotting analysis from Figure 4D.

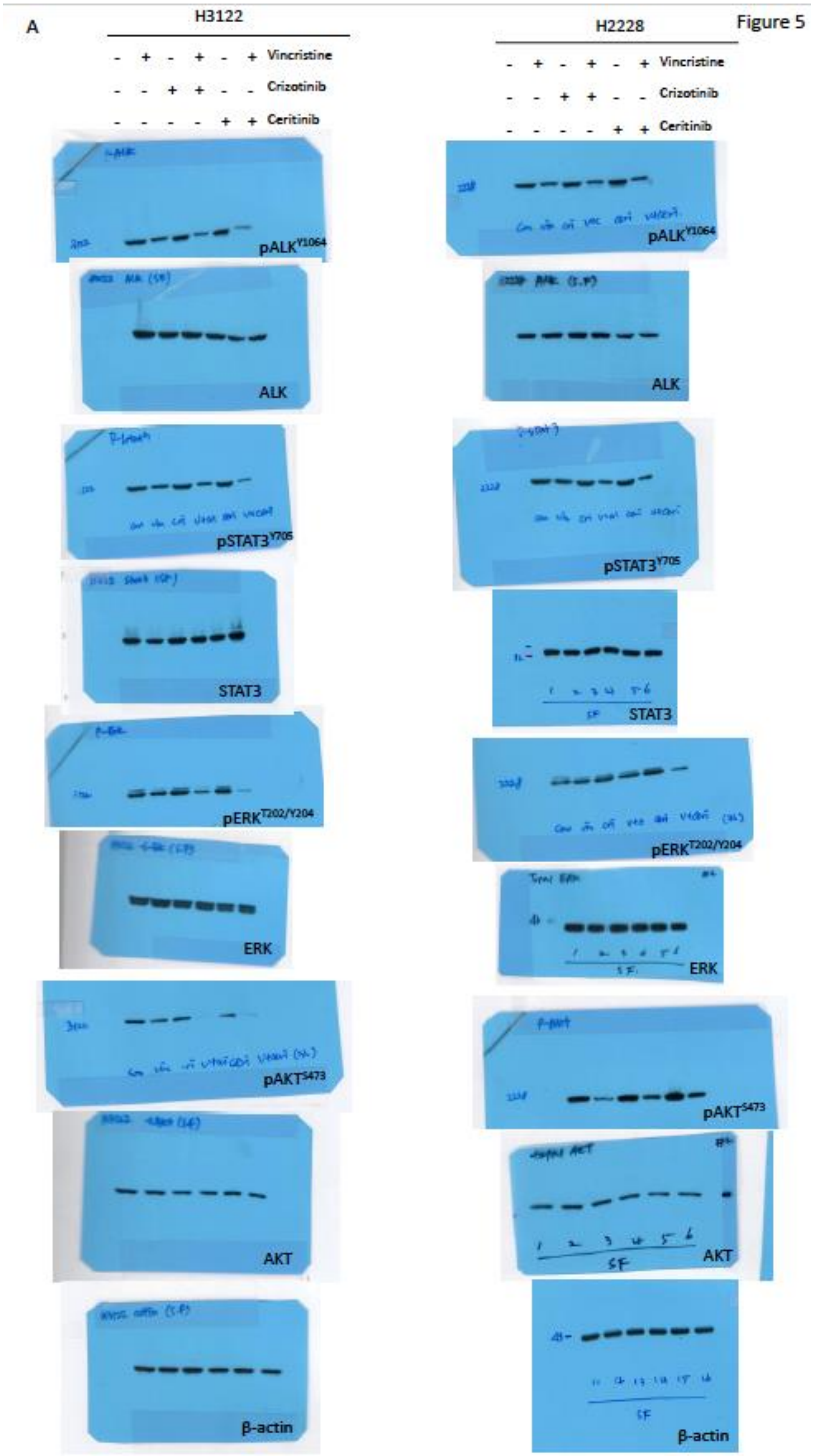

Figure S5 continued. Source data of western blotting analysis from Figure 5A.

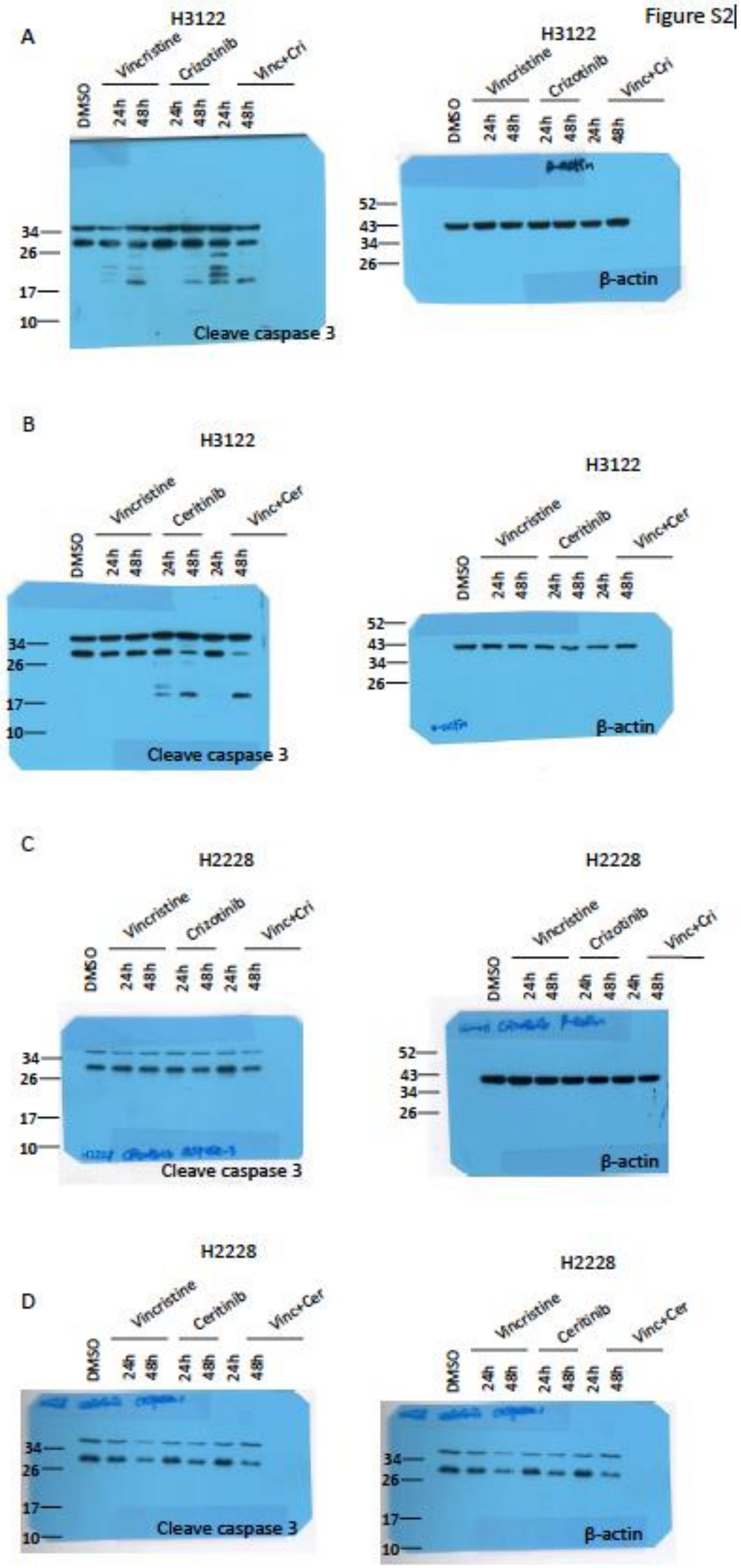

Figure S5 continued. Source data of western blotting analysis from Figure S2A-D.
